# Prevalence and factors associated with prediabetes among older adults in India: evidence from a national survey

**DOI:** 10.3389/fpubh.2026.1766812

**Published:** 2026-03-16

**Authors:** Rashita Ravi, Jeby Jose Olickal, Aajna Adoor, James Devasia, Kavumpurathu Raman Thankappan

**Affiliations:** 1 Department of Public Health, Amrita Institute of Medical Sciences, Amrita Vishwa Vidyapeetham, Kochi, Kerala, India; 2 Research Unit of Population Health, University of Oulu, Oulu, Finland; 3 Department of Preventive and Social Sciences, Jawaharlal Institute of Postgraduate Medical Education and Research (JIPMER), Puducherry, India

**Keywords:** prediabetes, risk factors, older adults, ageing population, prevalence

## Abstract

**Background:**

Prediabetes is a reversible intermediate stage of dysglycemia that substantially increases the risk of diabetes and cardiovascular disease. India is witnessing a rapid rise in the burden of prediabetes; however, national evidence on biomarker-verified prediabetes among older adults remains limited. The present study aimed to estimate the prevalence of prediabetes and identify its associated factors among older adults in India.

**Methods:**

We conducted a secondary cross-sectional analysis of wave one longitudinal aging study in India (LASI) data, a nationally representative survey of adults aged ≥45 years. A total of 58,386 participants with valid biomarker data on blood glucose levels were included. Prediabetes was defined as HbA1c 5.7–6.4%. Factors associated with prediabetes were examined using Poisson regression models to estimate adjusted prevalence ratios (aPRs) and 95% CIs.

**Results:**

The weighted prevalence of prediabetes among adults aged ≥45 years was 30.4% (95% CI: 29.5–31.2), and dysglycemia (prediabetes + diabetes) was 49.8% (95% CI: 48.9–50.7). Factors independently associated with higher prediabetes prevalence were age 60–74 years (aPR 1.22, 95% CI 1.15–1.29), ≥75 years (aPR 1.30, 95% CI 1.16–1.45), female sex (aPR 1.12, 95% CI 1.06–1.19), not currently working (aPR 1.08, 95% CI 1.02–1.15), other religions versus Hindu (aPR 1.12, 95% CI 1.02–1.22), no acute anemia in the past 2 years (aPR 1.12, 95% CI 1.02–1.22), overweight (aPR 1.56, 95% CI 1.41–1.73), and obesity (aPR 1.90, 95% CI 1.70–2.13). Prediabetes was most prevalent in Telangana (70.0%) and lowest in Sikkim (6.6%), while dysglycemia was highest in Telangana (78.0%), and lowest in Meghalaya (16.7%). Higher dysglycemia prevalence was independently associated with older age (60–74 and ≥75 years), urban residence, not currently working, family history of diabetes, hypertension, and excess adiposity, with the strongest associations observed for overweight (aPR 1.65, 95% CI 1.53–1.78) and obesity (aPR 1.86, 95% CI 1.72–2.02) (all *p* < 0.001).

**Conclusion:**

Prediabetes affects more than one in four older adults in India. The marked regional and socioeconomic disparities underscore the urgent need for population-specific, biomarker-based screening and prevention strategies targeting older adults to curb progression to diabetes and related complications.

## Introduction

Prediabetes represents a critical intermediate stage of dysglycemia characterized by blood glucose levels elevated beyond normal ranges yet remaining below the diagnostic threshold for diabetes mellitus (1). Globally, the burden of prediabetes has reached alarming proportions, with an estimated 634.8 million adults representing 12.0% of the global adult population having impaired glucose tolerance in 2024 (2, 3). Projections indicate that by 2050, cases will surge to 846.5 million, highlighting an accelerating epidemic that places immense strain on health systems and contributes to the growing global burden of non-communicable diseases (3, 4). In India, prediabetes imposes a substantial burden, affecting 136 million individuals; 15.3% of the population aged 20 years and above, with rising prevalence compounded by ageing, urbanization, and lifestyle transitions (5, 6).

The burden of elevated blood glucose is particularly pronounced among older adults. Age-related metabolic changes, accumulation of comorbidities, and physiological decline increase susceptibility to cardiovascular and kidney complications, functional limitations, and loss of independence (7–9). In addition, hypertension, obesity, and dyslipidemia commonly coexist with dysglycemia, further amplifying the risk (10, 11). Estimates suggest that among adults aged 45 years and above, a substantial proportion experience impaired glucose regulation, which is associated with increased healthcare utilization and long-term care needs (12–14). These factors highlight the growing public health challenge posed by elevated blood glucose in older populations and the importance of generating precise, population-level evidence.

Despite the recognized significance of prediabetes, most national-level studies in India have focused on younger adults and often lack biomarker-verified data, limiting understanding of prediabetes among adults aged 45 years and above (15, 16). The risk profile in this population is further complicated by the interplay of multimorbidity, polypharmacy, and interrelated metabolic, lifestyle, and sociodemographic factors (17, 18). Generating robust, population-level evidence is therefore essential to inform prevention strategies and reduce the long-term health and societal burden of prediabetes among older adults. The Longitudinal Ageing Study in India (LASI) provides nationally representative data on adults aged 45 years and above, including biomarker measurements and detailed health and socioeconomic information (19). The present study aimed to estimate the prevalence of prediabetes and to identify the factors associated with it among older adults in India.

## Methods

### Study design and data source

We conducted a secondary, cross-sectional analytical study using wave-1 data from the LASI-a nationally representative survey of adults aged ≥45 years. LASI wave-1 employed multistage, stratified, area-probability sampling (three stages in rural areas; four in urban areas) across Indian states/UTs; fieldwork was done during April 2017–December 2018, except Sikkim that was done during 2020–2021.

### Study participants

**Figure 1:**
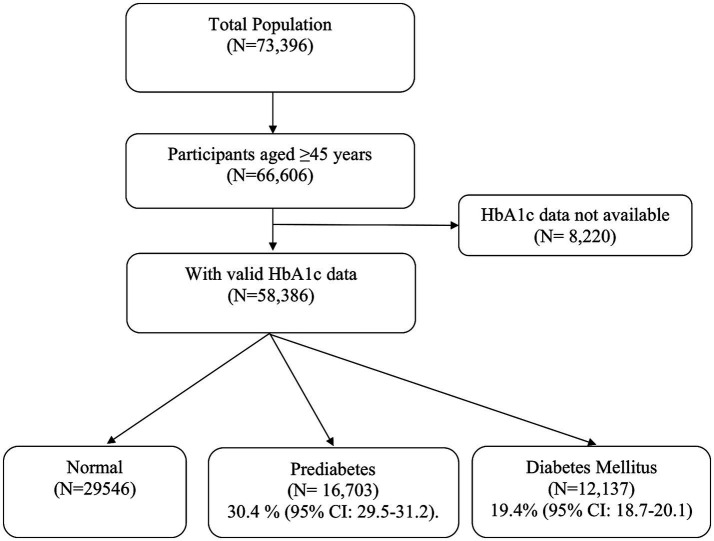
Selection of samples and prevalence estimates.

For this analysis, we first restricted the LASI wave 1 sample (*N* = 73,396) to respondents aged ≥45 years (*N* = 66,606). Of these, 58,386 participants had valid HbA_1_c measurements and were included to estimate the prevalence of prediabetes, and diabetes mellitus (Figure 1). For analyses of factors associated with prediabetes, we further excluded individuals with diabetes mellitus and those with missing covariate data, yielding a sample of 46,176 adults aged ≥45 years for analysis.

### Measurement

In LASI wave 1 (2017–18), glycated haemoglobin (HbA_1_c) was measured using dried blood spot (DBS) samples obtained from respondents aged 45 years and above. The collection process involved five finger-prick blood drops onto specialized filter paper cards, each uniquely bar-coded and dried for at least 4 h (typically overnight). These DBS specimens were transported to and analyzed at the Indian Council of Medical Research–National Institute of Translational Virology and AIDS Research (ICMR-NITVAR) laboratory in Pune. The HbA_1_c assay used 3.2 mm punches of the DBS, eluted and analyzed on a Roche Cobas Integra-400 Plus system and standardized to the National Glycohemoglobin Standardization Program (NGSP) or Diabetes Control and Complications Trial (DCCT) scale; internal quality controls and external quality assessment (EQAS) protocols were implemented. A calibration study using paired whole-blood and DBS samples confirmed a high correlation (r > 0.99) between DBS and venous measures, supporting the validity of the approach for large-scale population surveillance.

### Outcome

Participants were categorized based on HbA_1_c values, following the American Diabetes Association (ADA) 2024 criteria. Prediabetes was defined as HbA_1_c 5.7–6.4%. Dysglycemia referred to the combined burden of prediabetes and diabetes, (HbA_1_c ≥ 5.7% or self-reported diabetes).

### Exposures/covariates

Based on *a priori* determinants and availability in LASI, we included:

- Age (45–59, 60–74, ≥75; from dm005)
- Sex (male/female; dm003)
- Wealth [monthly per capita consumption expenditure (MPCE) quintile]
- Residence (rural/urban; residence)
- Religion (Hindu, Muslim, Christian, Others; dm010)
- Currently working (yes/no; “no” includes those retired, unemployed, or otherwise not engaged in paid employment)
- Family history (yes/no)
- Ever diagnosed hypertension (yes/no; ht002)
- Ever consumed any alcoholic beverages (yes/no; ht101)
- Ever smoked or used smokeless tobacco (yes/no; ht001)
- Body Mass Index (BMI)categorized as per WHO criteria: underweight (<18.5 kg/m^2^), normal (18.5–24.9 kg/m^2^), overweight (25.0–29.9 kg/m^2^), and obese (≥30.0 kg/m^2^); bmicat)
- History of acute disease or anemia in the past 2 years (yes/no; ht207)

### Weighting and representativeness

All analyses applied individual sampling weights (India DBS weights) to account for differential selection probabilities and non-response. Because replicate design variables are not available in the public-use LASI files, probability weights (pweights) with robust (Huber–White) standard errors were used.

### Statistical analysis

Analyses were performed using Stata version 14. Categorical variables were summarized as unweighted counts (*n*) and weighted percentages, while outcome variables were presented with 95% confidence intervals (CIs). For analyses of factors associated with prediabetes, individuals with diabetes mellitus were excluded to allow comparison between participants with prediabetes and those with normoglycaemic status. Participants with missing data on covariates included in the multivariable models were excluded using complete-case analysis, yielding a final analytic sample of 46,176 adults aged ≥45 years. Unadjusted prevalence ratios (uPRs) and their 95% CIs were estimated using log-binomial regression with probability weights (pweights). Adjusted prevalence ratios (aPRs) were obtained using multivariable log-binomial regression including relevant sociodemographic and health covariates. A *p*-value < 0.05 was considered statistically significant.

### Ethical considerations

LASI obtained national ethics approvals and informed consent from all participants; the public-use data are de-identified and available to researchers upon registration with Indian Institute of Population Sciences (IIPS). This secondary analysis used only anonymized data and did not require additional human-subjects review.

## Results

Over half were aged 45–59 years, and 53.6% were women. Most had primary-level education (58.7%), were married (74.8%), and lived in rural areas (65.9%). Hindus formed 73.2% of the sample. Over half were working (53.9%), 84.2% had a family history of diabetes and 28.8% reported hypertension. Alcohol and tobacco use were reported by 18.0 and 36.7%, respectively (Table 1).

**Table 1:** Sociodemographic and health-related characteristics (*N* = 58,386).

Variable	Category	*n*	Weighted %
Age in years	45–59	30,670	52.53
	60–74	22,040	37.75
	≥75	5,676	9.72
Gender	Male	27,118	46.45
	Female	31,268	53.55
Education	Up to primary	34,279	58.71
	Above primary	24,106	41.29
Wealth quintile	Poorest	11,581	19.84
	Poorer	11,740	20.11
	Middle	11,792	20.20
	Richer	11,808	20.22
	Richest	11,465	19.64
Place of residence	Rural	38,447	65.85
	Urban	19,939	34.15
Religion	Hindu	42,727	73.18
	Muslim	6,823	11.69
	Christian	5,783	9.90
	Others	3,053	5.23
Marital status	Currently married	43,667	74.79
	Currently not married	14,719	25.21
Currently working	Yes	31,458	53.88
	No	26,928	46.12
Family history	Yes	49,143	84.17
	No	9,243	15.83
Ever diagnosed hypertension	Yes	16,836	28.84
	No	41,532	71.16
Ever consumed any alcoholic beverages	Yes	10,513	18.02
	No	47,814	81.98
Ever smoked or used smokeless tobacco	Yes	21,416	36.73
	No	36,892	63.27
Body mass index (BMI)	Underweight	10,572	21.01
	Normal	30,261	51.52
	Overweight	12,746	20.53
	Obese	4,283	6.93
Anemia	Yes	2,334	4.58
	No	56,043	95.42

**Figure 2:**
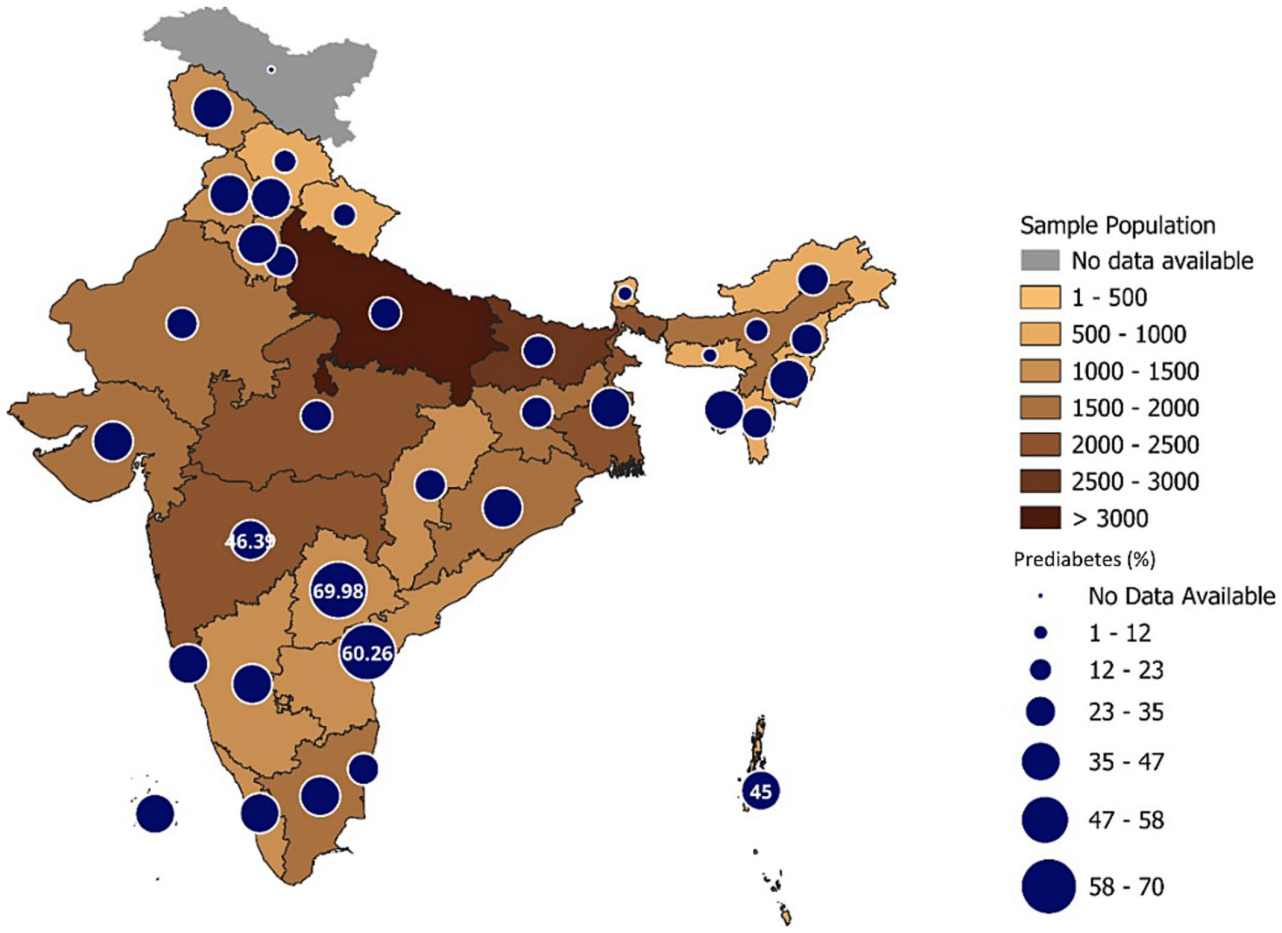
State-wise and gender wise distribution of prediabetes among older adults in India.

**Figure 3:**
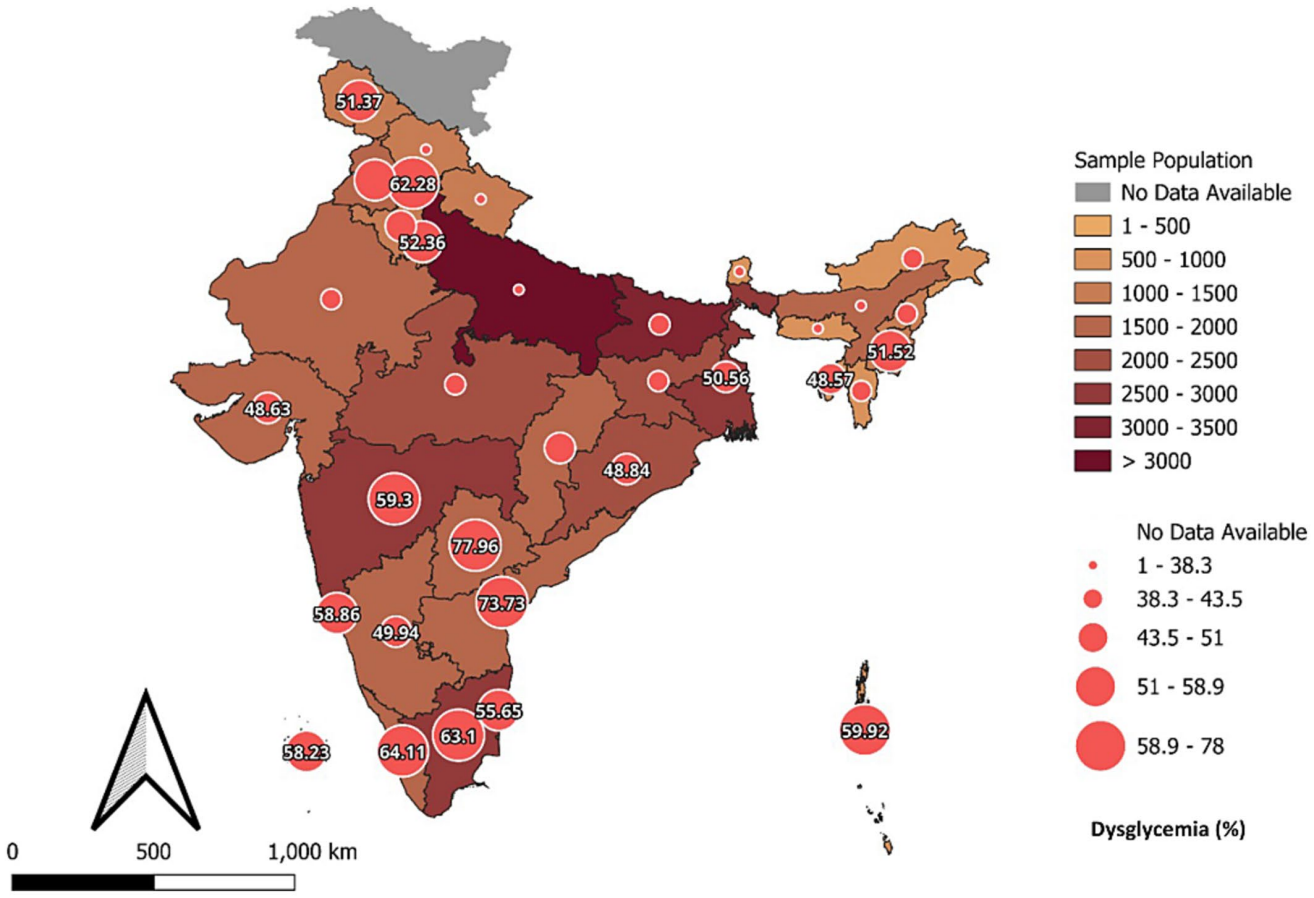
State-wise and gender wise distribution of dysglycemia among older adults in India.

The weighted prevalence of prediabetes among adults aged ≥45 years was 30.4% (95% CI: 29.5–31.2) (Figure 1) and the weighted prevalence of dysglycemia was 49.8% (95% CI: 48.9–50.7). Prediabetes was most prevalent in Telangana (70.0%) and lowest in Sikkim (6.6%) (Supplementary Table 1; Figure 2), while dysglycemia was highest in Telangana (78.0%), and lowest in Meghalaya (16.7%) (Supplementary Table 1; Figure 3).

Factors associated with prediabetes were examined among 45,817 adults aged ≥45 years without diabetes mellitus after excluding participants with missing covariate data. Factors independently associated with higher prediabetes prevalence were age 60–74 years (aPR 1.22, 95% CI 1.15–1.29; *p* < 0.001), ≥75 years (aPR 1.30, 95% CI 1.16–1.45; *p* < 0.001), female sex (aPR 1.12, 95% CI 1.06–1.19; *p* < 0.001), not currently working (aPR 1.08, 95% CI 1.02–1.15; *p* = 0.005), other religions versus Hindu (aPR 1.12, 95% CI 1.02–1.22; *p* = 0.012), no acute anemia in the past 2 years (aPR 1.12, 95% CI 1.02–1.22; *p* = 0.014), overweight (aPR 1.56, 95% CI 1.41–1.73; *p* < 0.001), and obesity (aPR 1.90, 95% CI 1.70–2.13; *p* < 0.001) (Table 2).

**Table 2:** Sociodemographic and health factors associated with prediabetes (*N* = 45,817).

Variable	*n*	Prediabetes *n* (%)	No prediabetes *n* (%)	uPR (95% CI)	*p*-value	aPR (95% CI)	*p*-value
Age (years)							
45–59	25,029	8,220 (32.84)	16,809 (67.16)	1	–	1	–
60–74	16,775	6,623 (39.48)	10,152 (60.52)	1.16 (1.10–1.23)	<0.001	1.22 (1.15–1.29)	**<0.001**
≥75	4,445	1,860 (41.84)	2,585 (58.16)	1.22 (1.10–1.35)	<0.001	1.30 (1.16–1.45)	**<0.001**
Sex							
Male	21,415	6,997 (32.67)	14,418 (67.33)	1	–	1	–
Female	24,834	9,706 (39.08)	15,128 (60.92)	1.23 (1.17–1.30)	<0.001	1.12 (1.06–1.19)	**<0.001**
Education							
Upto primary	28,552	10,193 (35.70)	18,359 (64.30)	1	–	–	–
Above primary	17,696	6,509 (36.78)	11,187 (63.22)	1.02 (0.95–1.09)	0.598	–	–
Wealth (MPCE quintile)							
Poorest	9,697	3,241 (33.42)	6,456 (66.58)	1	–	1	–
Poorer	9,600	3,391 (35.32)	6,209 (64.68)	1.02 (0.96–1.09)	0.502	1.01 (0.94–1.07)	0.852
Middle	9,376	3,334 (35.56)	6,042 (64.44)	1.08 (0.99–1.17)	0.101	1.03 (0.95–1.12)	0.489
Richer	9,087	3,378 (37.17)	5,709 (62.83)	1.09 (1.00–1.19)	0.038	1.04 (0.96–1.13)	0.355
Richest	8,489	3,359 (39.57)	5,130 (60.43)	1.12 (1.03–1.22)	0.008	1.03 (0.95–1.12)	0.414
Residence							
Rural	32,428	11,017 (33.97)	21,411 (66.03)	1	–	1	–
Urban	13,821	5,686 (41.14)	8,135 (58.86)	1.23 (1.15–1.33)	<0.001	1.07 (1.00–1.15)	0.051
Religion							
Hindu	34,062	12,343 (36.24)	21,719 (63.76)	1	–	1	–
Muslim	5,096	1,981 (38.87)	3,115 (61.13)	1.12 (1.04–1.21)	0.004	1.07 (1.00–1.15)	0.067
Christian	4,728	1,511 (31.96)	3,217 (68.04)	1.17 (0.89–1.53)	0.268	1.20 (0.90–1.59)	0.215
Others	2,363	868 (36.73)	1,495 (63.27)	1.20 (1.10–1.30)	<0.001	1.12 (1.03–1.22)	**0.012**
Marital status							
Currently married	34,673	12,159 (35.07)	22,514 (64.93)	1	–	–	–
Single	11,576	4,544 (39.25)	7,032 (60.75)	1.13 (1.06–1.20)	<0.001	–	–
Currently working							
Yes	22,669	7,349 (32.42)	15,320 (67.58)	1	–	1	–
No	23,580	9,354 (39.67)	14,226 (60.33)	1.27 (1.21–1.34)	<0.001	1.08 (1.02–1.15)	**0.005**
Family history of diabetes							
No	40,545	14,336 (35.36)	26,209 (64.64)	1	–	1	–
Yes	5,704	2,367 (41.50)	3,337 (58.50)	1.11 (0.99–1.24)	0.083	1.02 (0.92–1.12)	0.708
Ever diagnosed hypertension							
No	35,508	12,138 (34.18)	23,370 (65.82)	1	–	1	–
Yes	10,723	4,560 (42.53)	6,163 (57.47)	1.18 (1.11–1.25)	<0.001	1.01 (0.95–1.07)	0.829
Alcohol use (ever)							
Yes	8,674	2,657 (30.63)	6,017 (69.37)	1	–	1	–
No, never	37,533	14,030 (37.38)	23,503 (62.62)	1.24 (1.16–1.32)	<0.001	1.06 (0.99–1.14)	0.097
Tobacco (ever)							
Yes	17,918	5,879 (32.81)	12,039 (67.19)	1	–	1	–
No	28,280	10,806 (38.21)	17,474 (61.79)	1.20 (1.14–1.26)	<0.001	1.03 (0.98–1.09)	0.257
BMI category							
Underweight	9,767	2,879 (29.48)	6,888 (70.52)	1	–	1	–
Normal	24,944	8,305 (33.29)	16,639 (66.71)	1.15 (1.07–1.24)	<0.001	1.18 (1.09–1.27)	**<0.001**
Overweight	8,631	3,972 (46.02)	4,659 (53.98)	1.57 (1.42–1.74)	<0.001	1.56 (1.41–1.73)	**<0.001**
Obese	2,547	1,420 (55.75)	1,127 (44.25)	2.01 (1.82–2.21)	<0.001	1.90 (1.70–2.13)	**<0.001**
Had acute anemia in past 2 years							
Yes	1,904	636 (33.40)	1,268 (66.60)	1	–	1	–
No	44,337	16,064 (36.23)	28,273 (63.77)	1.11 (1.02–1.21)	0.022	1.12 (1.02–1.22)	**0.014**

uPR, Unadjusted Prevalence Ratio; aPR, Adjusted Prevalence Ratio; BMI, Body mass index, Row percentages are unweighted within category. aPR from a single Poisson regression with log link, robust SEs, and sampling weights (indiaindividualweight)., Analyses exclude participants with diabetes mellitus. The analytic sample was derived from adults aged ≥45 years with valid HbA_1_c measurements; complete-case analysis was applied for covariates. Bold values indicate statistically significant *p* value (*p* < 0.05) in the multivariable analysis.

Factors independently associated with higher dysglycemia prevalence were age 60–74 years (aPR 1.18, 95% CI 1.14–1.23; *p* < 0.001) and ≥75 years (aPR 1.20, 95% CI 1.11–1.30; *p* < 0.001), urban residence (aPR 1.11, 95% CI 1.06–1.16; *p* < 0.001), not currently working (aPR 1.12, 95% CI 1.08–1.16; *p* < 0.001), family history of diabetes (aPR 1.13, 95% CI 1.07–1.19; *p* < 0.001), and hypertension (aPR 1.17, 95% CI 1.12–1.21; *p* < 0.001). Higher prevalence was also observed among individuals with overweight (aPR 1.65, 95% CI 1.53–1.78; *p* < 0.001), and obesity (aPR 1.86, 95% CI 1.72–2.02; *p* < 0.001) (Table 3).

**Table 3:** Sociodemographic and health factors associated with dysglycemia (*N* = 58,386).

Variable	*n*	Dysglycemia *n* (%)	Normal *n* (%)	uPR (95% CI)	*p*-value	aPR (95% CI)	*p*-value
Age (years)							
45–59	30,670	13,861 (45.19)	16,809 (54.81)	1	–	1	–
60–74	22,040	11,888 (53.94)	10,152 (46.06)	1.17 (1.13–1.22)	<0.001	1.18 (1.14–1.23)	**<0.001**
≥75	5,676	3,091 (54.46)	2,585 (45.54)	1.16 (1.08–1.25)	<0.001	1.20 (1.11–1.30)	**<0.001**
Sex							
Male	27,118	12,700 (46.83)	14,418 (53.17)	1	–	1	–
Female	31,268	16,140 (51.62)	15,128 (48.38)	1.14 (1.10–1.18)	<0.001	0.99 (0.95–1.04)	0.702
Education							
Upto primary	34,279	15,920 (46.44)	18,359 (53.56)	1	–	1	–
Above primary	24,106	12,919 (53.59)	11,187 (46.41)	1.13 (1.08–1.17)	<0.001	0.99 (0.95–1.04)	0.726
MPCE quintile							
Poorest	11,581	5,125 (44.25)	6,456 (55.75)	1	–	1	–
Poorer	11,740	5,531 (47.11)	6,209 (52.89)	1.01 (0.97–1.06)	0.583	0.99 (0.94–1.03)	0.589
Middle	11,792	5,750 (48.76)	6,042 (51.24)	1.07 (1.01–1.13)	0.024	1.01 (0.95–1.06)	0.838
Richer	11,808	6,099 (51.65)	5,709 (48.35)	1.12 (1.06–1.18)	<0.001	1.03 (0.97–1.09)	0.276
Richest	11,465	6,335 (55.26)	5,130 (44.74)	1.20 (1.13–1.28)	<0.001	1.04 (0.98–1.10)	0.209
Residence							
Rural	38,447	17,036 (44.31)	21,411 (55.69)	1	–	1	–
Urban	19,939	11,804 (59.20)	8,135 (40.80)	1.33 (1.28–1.39)	<0.001	1.11 (1.06–1.16)	**<0.001**
Religion							
Hindu	42,727	21,008 (49.17)	21,719 (50.83)	1	–	1	–
Muslim	6,823	3,708 (54.35)	3,115 (45.65)	1.10 (1.04–1.15)	<0.001	1.04 (0.99–1.09)	0.120
Christian	5,783	2,566 (44.37)	3,217 (55.63)	1.14 (0.99–1.30)	0.063	1.14 (0.97–1.35)	0.116
Others	3,053	1,558 (51.03)	1,495 (48.97)	1.14 (1.07–1.21)	<0.001	1.05 (0.99–1.11)	0.132
Marital status							
Currently married	43,667	21,153 (48.44)	22,514 (51.56)	1	–	1	–
Single	14,719	7,687 (52.23)	7,032 (47.77)	1.10 (1.05–1.14)	<0.001		
Currently working							
No	31,458	17,232 (54.78)	14,226 (45.22)	1.29 (1.25–1.34)	<0.001	1.12 (1.08–1.16)	**<0.001**
Yes	26,928	11,608 (43.11)	15,320 (56.89)	1	–	1	–
Family history of diabetes							
No	49,143	22,934 (46.67)	26,209 (53.33)	1	–	1	–
Yes	9,243	5,906 (63.90)	3,337 (36.10)	1.28 (1.20–1.36)	<0.001	1.13 (1.07–1.19)	**<0.001**
Ever diagnosed hypertension							
Yes	16,836	10,673 (63.39)	6,163 (36.61)	1.39 (1.34–1.45)	<0.001	1.17 (1.12–1.21)	**<0.001**
No	41,532	18,162 (43.73)	23,370 (56.27)	1	–	1	–
Ever consumed alcohol							
Yes	10,513	4,496 (42.77)	6,017 (57.23)	1	–	1	–
No, never	47,814	24,311 (50.84)	23,503 (49.16)	1.21 (1.15–1.26)	<0.001	1.05 (1.00–1.11)	0.032
Ever used tobacco (smoked/smokeless)							
Yes	21,416	9,377 (43.79)	12,039 (56.21)	1	–	1	–
No	36,892	19,418 (52.63)	17,474 (47.37)	1.22 (1.18–1.27)	<0.001	1.06 (1.02–1.10)	**0.003**
BMI category							
Underweight	10,572	3,684 (34.85)	6,888 (65.15)	1	–	1	–
Normal	30,261	13,622 (45.02)	16,639 (54.98)	1.30 (1.22–1.38)	<0.001	1.27 (1.20–1.35)	**<0.001**
Overweight	12,746	8,087 (63.45)	4,659 (36.55)	1.79 (1.67–1.91)	<0.001	1.65 (1.53–1.78)	**<0.001**
Obese	4,283	3,156 (73.69)	1,127 (26.31)	2.16 (2.03–2.31)	<0.001	1.86 (1.72–2.02)	**<0.001**
Anemia							
Yes	2,334	1,066 (45.67)	1,268 (54.33)	1	–	1	–
No	56,043	27,770 (49.55)	28,273 (50.45)	1.03 (0.95–1.11)	0.520	1.02 (0.95–1.11)	0.579

uPR, Unadjusted Prevalence Ratio; aPR, Adjusted Prevalence Ratio; BMI, Body mass index, Row percentages are unweighted within category. aPR from a single Poisson regression with log link, robust SEs, and sampling weights (indiaindividualweight). Bold values indicate statistically significant *p* value (*p* < 0.05) in the multivariable analysis.

## Discussion

The present analysis demonstrates a high prevalence of prediabetes (30.6%) and dysglycemia (50.3%) among adults aged ≥ 45 years in India, signalling a major public-health concern. These values are somewhat higher than national estimates reported by the ICMR-INDIAB study, which found a diabetes prevalence of 7.5% and prediabetes of 15–33%, depending on diagnostic criteria (20, 21). This difference is expected since the LASI cohort includes older individuals, in whom impaired glucose regulation is more common (22). Compared with previous national studies over the past decade, there is evidence of an increasing burden of prediabetes in middle-aged and older adults, reflecting the rapid epidemiological transition in India (5, 20, 23). The findings reinforce that India now carries the world’s second-largest burden of diabetes and prediabetes, with over 230 million affected adults (24).

Our reliance on glycated haemoglobin from dried-blood-spot assays offers an operational advantage but requires cautious interpretation. ICMR-INDIAB and earlier work have shown that using HbA_1_c alongside OGTT substantially increases apparent dysglycemia prevalence because of iron-deficiency anemia, which can spuriously elevate HbA_1_c (25, 26). National guidelines therefore recommend combining glucose-based measures with HbA_1_c for diagnosis (27). Future LASI biomarker rounds should adjust HbA_1_c values for ferritin or haemoglobin levels to refine population estimates (26, 27).

Age-related progression of dysglycemia was evident; participants aged 60–74 years had 22% higher risk and those aged ≥ 75 years had 30% higher risk compared with 45–59 years. This is consistent with nationwide trends demonstrating exponential rises in glucose intolerance after midlife (21, 28). Female predominance (aPR 1.12) mirrors patterns observed across South Asia, where post-menopausal weight gain, abdominal adiposity, and lower physical-activity levels contribute to insulin resistance (29, 30). These sex-age interactions underscore the need for targeted screening beginning at 45 years and specific outreach to older women (31), who are often missed in primary-care screening.

Socio-economic correlates were equally important. Individuals not currently working had greater prediabetes prevalence (aPR 1.08), reflecting the metabolic consequences of low physical activity (32). ICMR-INDIAB reported that nearly half of Indian adults were physically inactive, with particularly high rates among women and urban residents (33). Urbanization brings dietary shifts towards calorie-dense foods and prolonged sedentary time, both independently linked to higher glycaemic indices (34). Integrating structured physical-activity modules into Health & Wellness Centres and promoting neighborhood-level exercise programs could mitigate this risk (35).

Religious differences observed in our data likely reflect underlying regional or cultural heterogeneity rather than faith itself. States with larger proportions of non-Hindu populations; such as Kerala, Telangana, and Andhra Pradesh; also report higher diabetes prevalence in national surveys (20, 36). Dietary customs, including higher refined-carbohydrate intake and certain cooking-oil patterns, contribute to these inter-state disparities (37). Hence, culturally tailored nutrition counselling, adapted to local cuisines and beliefs, is essential to enhance adherence (38).

Adiposity exhibited the strongest association: overweight (aPR 1.56) and obesity (aPR 1.90) nearly doubled prediabetes risk. This aligns with the “Asian-Indian phenotype” characterized by excess visceral fat and insulin resistance at comparatively low BMI values (39, 40). National data indicate that over 40% of Indian adults are abdominally obese (41). Even modest 5–7% weight reduction through lifestyle change can halve diabetes incidence among high-risk individuals (14). These results reaffirm that weight management must remain the cornerstone of India’s diabetes-prevention agenda (7).

Dysglycemia also clustered with family history of diabetes and hypertension, reflecting aggregation of metabolic risk (42, 43). ICMR-INDIAB found hypertension prevalence exceeding 30%, which doubles the probability of developing diabetes (44). Such clustering of obesity, hypertension, and hyperglycaemia underscores the need for integrated screening and management under the National Programme for Prevention and Control of Non-Communicable Diseases (NP-NCD) (45). Evidence from large multi-state studies supports simultaneous detection of blood pressure, lipids, and glucose as a cost-effective approach (46).

Marked geographic heterogeneity was evident. Prediabetes prevalence exceeded 60% in Telangana and Andhra Pradesh, with dysglycemia surpassing 70% in southern states; closely mirroring the ICMR-INDIAB gradient showing advanced epidemic stages in southern India (20, 47). Conversely, several northern and eastern states remain at earlier transition stages (21). These dual epidemic demands state-specific strategies: southern states must prioritise secondary prevention and complication control, while northern states focus on primary prevention through community-based lifestyle modification (48). Interstate difference in prediabetes among adults aged 20 years and above ranged from 6.8% in Mizoram to 31.3% in Sikkim as reported in a recent Indian study in the Lancet Diabetes and endocrinology (49). The larger interstate difference in the older adults in the LASI study could be due to various factors such as levels of urbanization, obesity prevalence, dietary practices, and physical activity patterns are likely contributors to these variations. At the same time, methodological factors may also play a role. Fieldwork for LASI in Sikkim was conducted later than in most other states, and state-level sample sizes in some northeastern regions are relatively small, which may increase variability in prevalence estimates. In addition, reliance on HbA_1_c-based assessment may introduce some degree of detection bias. Despite policy efforts, control rates remain low; fewer than 7% of adults with known diabetes achieve recommended glycaemic, lipid, and blood-pressure targets (50). Scaling up integrated chronic-care models, especially at primary-care level, could bridge this gap. Use of digital tracking, follow-up calls, and decentralised drug procurement within Ayushman Bharat Health & Wellness Centres can improve adherence (43, 50, 51). Moreover, evidence shows that the effectiveness of lifestyle interventions varies across prediabetes phenotypes, with significant risk reduction (52), underscoring the need for phenotype-specific prevention strategies.

Although our study is cross-sectional, its strengths include a large nationally representative sample, weighted estimates, and alignment with other high-quality national datasets. However, this study has certain limitations. The cross-sectional design limits causal inference regarding the temporal relationships between identified risk factors and prediabetes. HbA_1_c was measured using dried blood spot samples which, although validated, may be subject to minor variability related to sample storage and environmental conditions. Although the LASI dataset includes self-reported information on physical activity and selected dietary practices, these variables were not assessed using standardized or quantitative measures. Physical activity was captured through broad frequency-based questions without detailed information on intensity or duration, and dietary data were limited to coarse food-frequency indicators without portion size or nutrient-level assessment. Given the older study population and the potential for recall error and misclassification, these variables were not included in the adjusted models, and some degree of residual confounding related to lifestyle factors cannot be entirely excluded. HbA_1_c measurements were unavailable for a subset of eligible participants due to biomarker collection constraints, and a smaller proportion of participants were excluded from multivariable analyses because of missing covariate data. While survey weights were applied to mitigate non-response and support population-representative inference, some degree of residual selection bias cannot be completely ruled out. Potential interaction effects or subgroup heterogeneity, such as effect modification by age or obesity, were not examined; exploring these aspects may provide further insights into high-risk subpopulations in future research.

## Conclusion

Nearly one-third of older Indian adults have prediabetes, and half of them have dysglycemia, with risk highest among women, urban residents, and those overweight or obese. These results mirror national findings and reinforce the need for comprehensive, state-specific strategies that integrate screening, weight management, and early intervention within India’s expanding NCD framework. Prediabetes status is a window of opportunity for targeted intervention focusing on healthy diet and physical activity to prevent or delay progression to diabetes. The Indian national program for prevention and control of NCDs (NP-NCD) needs to include detection of prediabetes at least for older adults so that targeted intervention can be provided.

## Data Availability

The data analyzed in this study were obtained from the Longitudinal Ageing Study in India (LASI). The dataset is publicly available upon registration with the International Institute for Population Sciences (IIPS), Mumbai. Researchers can apply for access through the LASI website (https://lasi-india.org).
