# Supplementary material for: Prevalence and factors associated with prediabetes among older adults in India: evidence from a national survey
Source: Front Public Health. 2026 Mar 16;14:1766812. doi: 10.3389/fpubh.2026.1766812 (PMC13033546; doi:10.3389/fpubh.2026.1766812)
Supplement: Supplementary file 1 [file Data_Sheet_1.pdf]

Supplementary table 1: State-wise prevalence of prediabetes and dysglycemia

State	Prediabetes_%	Dysglycemia_%
Jammu and Kashmir	41.1	51.37
Himachal Pradesh	19.76	32.32
Punjab	36.06	52.36
Chandigarh	41.24	62.28
Uttarakhand	21.39	33.47
Haryana	41.47	50.78
Delhi	32.32	52.36
Rajasthan	33.79	43
Uttar Pradesh	28.42	38.04
Bihar	31.34	41.14
Sikkim	6.65	27.14
Arunachal Pradesh	32.78	40.64
Nagaland	32.09	39.19
Manipur	41.64	51.52
Mizoram	32.19	41.97
Tripura	39.7	48.57
Meghalaya	10.28	16.75
Assam	23.18	32.04
West Bengal	37.65	50.56
Jharkhand	33.56	43.47
Odisha	38.61	48.84
Chhattisgarh	33.99	43.54
Madhya Pradesh	29.37	38.39
Gujarat	37.03	48.63
Daman and Diu	35.38	52.91
Dadra and Nagar Haveli	46.9	55.57
Maharashtra	46.39	59.3
Andhra Pradesh	60.26	73.73
Karnataka	37.03	49.94
Goa	39.17	58.86
Lakshadweep	35.83	58.23
Kerala	41.81	64.11
Tamil Nadu	43.72	63.1
Puducherry	30.28	55.65
Andaman and Nicobar	45	59.92
Telangana	69.98	77.96
